# Bridging the mind and body: exploring venous thromboembolism in psychiatric inpatients

**DOI:** 10.1177/10398562251353668

**Published:** 2025-06-29

**Authors:** Ryan RD Chan, Emma Hamid, Thuy Le, Mariam Alaverdashvili, Annabelle Wanson, Katelyn Halpape

**Affiliations:** College of Pharmacy and Nutrition, University of Saskatchewan, Saskatoon, SK, Canada; Department of Psychiatry, College of Medicine, 7235 University of Saskatchewan, Saskatoon, SK, Canada; College of Pharmacy and Nutrition, 7235 University of Saskatchewan, Saskatoon, SK, Canada

**Keywords:** venous thromboembolism, inpatient psychiatry, mechanical restraints, electroconvulsive therapy, catatonia

## Abstract

**Objective:**

This study aimed to identify factors associated with venous thromboembolism (VTE) diagnosis in psychiatric inpatients in Saskatoon, Saskatchewan, Canada.

**Methods:**

We conducted a retrospective case-control chart review of patients admitted to the Dube Centre for Mental Health from January 2007 to December 2021. Cases were individuals aged 18 years and older who received anticoagulation for VTE treatment. Controls were randomly selected, with case-to-control ratio 1:4, from patients with a discharge diagnosis not including VTE. Data were analyzed using descriptive analysis, univariate, followed by multivariable logistic regression analysis to identify factors associated with VTE diagnosis.

**Results:**

A total of 32 VTE and 159 non-VTE patients were included. The mean age of VTE patients was 52 years (standard deviation [SD] = 19.7), 65.6% were female, and 65.6% had no previous VTE. Comorbidities including cancer (adjusted odds ratio [AOR] = 51.83; *p* = .004), cardiovascular conditions (AOR = 7.83; *p* = .01), and insomnia (AOR = 88.79; *p* = .01); psychiatric-specific interventions such as electroconvulsive therapy (AOR = 21.10; *p* < .001) and mechanical restraints (AOR = 12.65; *p* = .004); and acute medical diagnoses (AOR = 8.56; *p* = .01) were independently associated with developing VTE.

**Conclusions:**

Psychiatric inpatients have unique factors that increase the likelihood of developing VTE. Further research with a larger sample size and multicenter design is needed.

Venous thromboembolism (VTE), comprising deep vein thrombosis (DVT) and pulmonary embolism (PE), are preventable complications of inpatient psychiatric care.^1^ The incidence of VTE in psychiatric inpatients ranges from 2.3 to 25.3%.^2,3^ Well-established guidelines exist for VTE prevention in medical environments; however, specific protocols for use in psychiatry are not established.^4^

VTE development is influenced by non-modifiable risk factors such as gender, ethnicity, and age as well as by modifiable risk factors such as surgery, malignancy, trauma, pregnancy, and certain medications.^5^ Psychiatric inpatients are at increased risk of VTE due to multiple factors, including excessive sedation, antipsychotic use, catatonia, electroconvulsive therapy (ECT), and mechanical restraint use.^6,7^

While local and national protocols for thromboprophylaxis in adult hospitalized patients exist in Australasia, the absence of psychiatric-specific VTE risk assessment models (RAMs) may contribute to an increased risk of VTE among psychiatric inpatients due to suboptimal identification of at risk patients.^8–11^ VTE identification in psychiatric patients may be further delayed due to patient difficulty in communicating symptoms and erroneous attribution of symptoms to a psychosomatic cause.^12^ Some studies have evaluated pharmacological thromboprophylaxis for psychiatric inpatients; however, most studies were too small to determine which patients should receive anticoagulation.^13,14^

This study was undertaken to inform the development of a VTE prophylaxis RAM for psychiatric inpatients admitted to the Dube Centre for Mental Health (DCMH) in Saskatoon, Saskatchewan, Canada. More specifically, this study aimed to identify psychiatric inpatients who developed VTE, describe their characteristics, and identify factors contributing to VTE.

## Methods

### Study design and setting

This study involved a retrospective case-control chart review of psychiatric inpatients admitted to the DCMH from January 2007 to December 2021. Research ethics approval was obtained from the University of Saskatchewan Research Ethics Board (USask-REB-3348) and Operational Approval from the Saskatchewan Health Authority (OA-UofS-3348).

Data were reviewed and coded by RRDC using standard coding tools. To ensure the accuracy of the data collection, total inter-rater reliability was examined on 10% of all charts with >90% agreement by RRDC and EH, who individually screened, reviewed, and coded the data to achieve consensus on the accuracy of collection.

### Case selection

All patients that experienced a VTE during their psychiatric hospitalization were identified using the inpatient pharmacy software (BDM-Centricity Pharmacy Software) to run a drug utilization report for all patients who received an anticoagulant. The anticoagulants considered were heparin, dalteparin, enoxaparin, tinzaparin, warfarin, fondaparinux, dabigatran, rivaroxaban, apixaban, and edoxaban at a dose that would treat acute VTE. Patient charts were selected for inclusion by screening for individuals aged 18 years and older who received anticoagulation for VTE treatment. Charts were excluded if the patient received anticoagulation for other indications (e.g., atrial fibrillation) or if the patient was under the age of 18 years. Charts missing relevant data, such as VTE diagnostic imaging (e.g., computerized tomography scan) or progress notes were also excluded.

### Control selection

**Figure 1.**
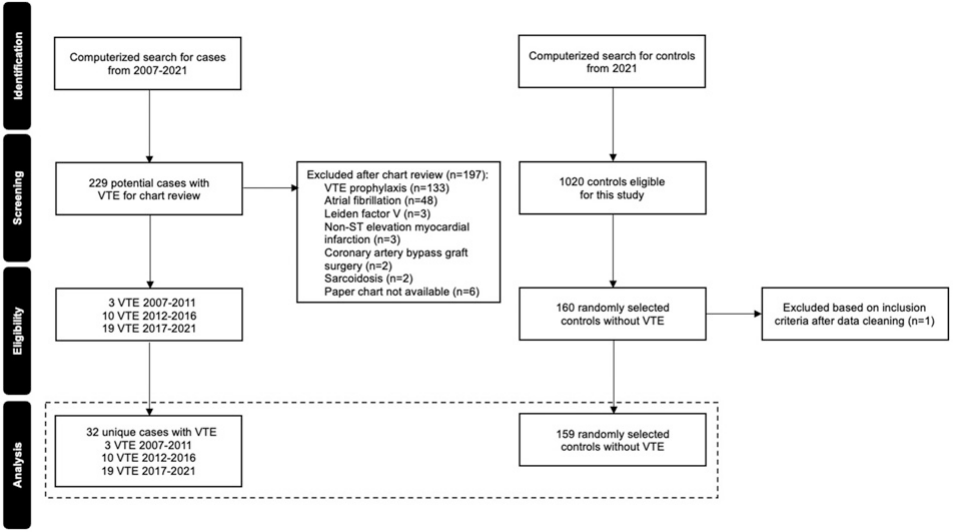
Flow chart showing the process of selection of cases with venous thromboembolism (VTE) and controls.

Based on an estimated case sample of 40 patients, four non-VTE controls per case patient (control *n* = 160) were randomly selected from the electronic health record (Sunrise Clinical Manager (SCM) Register) based on the following criteria: individuals aged 18 years and older, psychiatric hospitalization between January to December 2021, and a discharge diagnosis not including VTE. The final sample of controls included 159 participants as one patient did not meet the inclusion criteria during data cleaning (Figure 1).

### Data

The following information was obtained from the patient charts: age, sex, ethnicity, weight, height, length of hospital stay, chief complaint, past psychiatric and medical comorbidities, active substance use, inpatient medications, psychiatric and medical comorbidities at discharge, and patient outcomes. For case patients, data on known VTE risk factors and diagnostic imaging, the type and location of VTE, and interventions and treatments used in hospital were also collected. Data elements were extracted based on variables commonly identified in the literature as relevant to the risk, diagnosis, and management of VTE, as well as to characterize the patient population and clinical course.

### Data analysis

Descriptive analysis involved frequency calculations (e.g., count, percentage, mean, and median) of studied variables. Contingency tables were formed for each variable. For continuous variables, data distribution was checked using the skewness test. Means and medians were then calculated for symmetric and asymmetric continuous variables, respectively. Differences between VTE and non-VTE patients were examined using Pearson’s chi-square test or alternative (i.e., Fisher’s Exact test or Likelihood ratio test if the percentage of cells with expected count less than 5 was >20%) for categorical variables, student *t* test for symmetric continuous variables and Mann–Whitney U test for asymmetric continuous variables.

To identify potential factors associated with VTE diagnosis, univariate, followed by multivariable logistic regression analysis was performed. Variables significantly associated with VTE diagnosis in univariate analysis were included in the multivariable logistic regression analysis. The selection of variables into a multivariable logistic regression model was also based on prior knowledge and biological plausibility, regardless of their *p*-values. Sex, age, and variables with *p*-value <.05 after a stepwise backward elimination were retained in the final multivariable logistic regression model. The validity of the final model was checked based on the magnitude of the adjusted odds ratio (AOR), 95% confidence intervals (95% CIs), Wald chi-square statistics, and adjusted R squares. Results were considered statistically significant if the two-tailed level of significance was *p* < .05. All analyses were performed using SPSS-28.

## Results

### Demographics and clinical profile of the study population

A total of 32 VTE and 159 non-VTE patients were included in this study. Females accounted for 60.0% (*n* = 115) of the total study sample (Table 1). The mean age of all patients included in the study was 38.9 years (SD = ±16.7 years); however, VTE patients were older as compared with non-VTE patients (52.3 years vs 36.1 years; *p* < .001). There were no significant differences in sex, body mass index, and hormonal medications (e.g., hormonal contraceptives, hormone replacement therapy) between groups.

**Table 1.** Baseline characteristics and comparison between VTE and Non-VTE patients.

Variable	VTE (*n* = 32)	Non-VTE (*n* = 159)	*p*-value
Age (years)	52.3 ± 19.7	36.1 ± 14.6	<0.001
Sex, female	21 (65.6%)	94 (59.1%)	0.49
Ethnicity, Caucasian	27 (84.4%)	108 (67.9%)	0.11
Cigarette smoking			0.001
Yes	13 (40.6%)	112 (70.4%)	
No	19 (59.4%)	47 (29.6%)	
Medical comorbidities			
Autoimmune	5 (15.6%)	15 (9.4%)	0.34
Cancer	4 (12.5%)	2 (1.3%)	0.01
Cardiovascular	23 (71.9%)	22 (13.8%)	<0.001
Gastrointestinal	14 (43.8%)	26 (16.4%)	<0.001
Gynecological	6 (18.8%)	13 (8.2%)	0.10
Infectious diseases	1 (3.1%)	6 (3.8%)	0.99
Neurological	9 (28.1%)	24 (15.1%)	0.08
Respiratory	13 (40.6%)	18 (11.3%)	<0.001
Total number of medical comorbidities (median and IQR)	4.0 (2.3–6.0)	1.0 (0.0–2.0)	<0.001
Psychiatric comorbidities			
ADHD	3 (9.3%)	25 (15.7%)	0.37
Anxiety disorders	22 (68.8%)	67 (42.1%)	0.04
Autism spectrum disorder	1 (3.1%)	0 (0.0%)	0.17
Eating disorders	0 (0.0%)	5 (3.1%)	0.59
Insomnia	6 (18.8%)	2 (1.3%)	<0.001
Major depressive disorder	15 (46.9%)	63 (39.6%)	0.45
Mood disorders	20 (62.5%)	23 (14.5%)	<0.001
Personality disorders	6 (18.8%)	26 (16.4%)	0.27
Schizophrenia	8 (25.0%)	42 (26.4%)	0.88
Substance use disorders	5 (15.6%)	47 (29.6%)	0.15
Suicidal ideation	9 (28.1%)	36 (22.6%)	0.51
Total number of psychiatric comorbidities (median and IQR)	3.0 (2.0-4.8)	2.0 (1.0-3.0)	0.01

VTE: venous thromboembolism; IQR: interquartile range; ADHD: attention-deficit/hyperactivity disorder.

A notable difference was seen in the median length of hospital stay between VTE (39.5 days; IQR = 22.3–72.0) and non-VTE patients (9.0 days; IQR = 6.0–17.0) (Table 2). VTE patients were significantly more likely to have concurrent medical and psychiatric comorbidities than non-VTE patients (22.0% vs 2.0%; *p* < .001). There were no statistically significant differences overall in the use of psychotropics between VTE and non-VTE patients.

**Table 2.** Clinical characteristics of psychiatric inpatients during admission and at discharge.

Variable	VTE (*n* = 32)	Non-VTE (*n* = 159)	*p*-value
Length of stay (days) (median and IQR)	39.5 (22.3–72.0)	9.0 (6.0–17.0)	<0.001
Psychiatric-specific interventions			
Electroconvulsive therapy	16 (50.0%)	13 (8.2%)	<0.001
Mechanical restraints	6 (18.8%)	5 (3.1%)	0.004
Acute medical diagnosis during psychiatric admission			<0.001
Yes	26 (81.3%)	53 (33.3%)	
No	6 (18.8%)	106 (66.7%)	
Medications received during psychiatric admission			
Antidepressants	25 (78.1%)	110 (69.2%)	0.31
Antipsychotics	31 (96.9%)	148 (93.1%)	0.69
Antiseizure	7 (21.9%)	28 (17.6%)	0.57
Benzodiazepines	27 (84.4%)	143 (89.9%)	0.36
Hormonal therapy	6 (18.8%)	18 (11.3%)	0.25
Mood stabilizers	15 (46.9%)	53 (33.3%)	0.14
Opioids	4 (12.5%)	11 (6.9%)	0.29
Sedatives	20 (62.5%)	73 (45.9%)	0.09
Psychiatric diagnosis at discharge			
ADHD	2 (6.3%)	12 (7.5%)	0.80
Anxiety disorders	13 (40.6%)	71 (44.7%)	0.74
Catatonia	5 (15.6%)	3 (1.9%)	0.004
Major depressive disorder	11 (34.4%)	47 (29.6%)	0.59
Major neurocognitive disorder	1 (3.1%)	0 (0.0%)	0.17
Mood disorders	16 (50.0%)	42 (26.4%)	0.02
Personality disorders	5 (15.6%)	35 (22.0%)	0.46
Schizophrenia	9 (28.1%)	64 (40.3%)	0.29
Polysubstance use disorder	2 (6.3%)	98 (61.6%)	<0.001
Suicidal ideation	3 (9.4%)	0 (0.0%)	0.004
Died during admission	1 (3.1%)	0 (0.0%)	0.17

VTE: venous thromboembolism; IQR: interquartile range; ADHD: attention-deficit/hyperactivity disorder.

Most notably, discharge diagnoses differed significantly between the two groups. VTE patients were more often discharged with suicidal ideation (9.4% vs 0.0%; *p* = .004) and bipolar I disorder (37.5% vs 17.0%; *p* = .01), while non-VTE patients were more often discharged with polysubstance use disorder (61.6% vs 6.3%; *p* < .001).

### Profile of VTE patients

The median days from admission to VTE diagnosis was 9.5 (IQR 4.0-21.8 days). Among VTE patients, nine experienced a DVT (28.1%), 15 had a PE (46.9%), and eight had a combined DVT and PE (25.0%). Eleven patients (34.4%) had a history of VTE. Twenty-eight patients (87.5%) had symptomatic VTE (e.g., chest pain, shortness of breath, leg swelling, and cramping). Additionally, 26 VTE patients (81.3%) had an acute medical diagnosis (separate from VTE) during hospitalization, with 12 VTE events (46.2%) identified during the COVID-19 pandemic.

### Factors associated with VTE diagnosis

Results from the multivariable logistic regression analysis indicate that comorbidities, including cancer (AOR = 51.83; *p* = .004), cardiovascular conditions (AOR = 7.83; *p* = .01), and insomnia (AOR = 88.79; *p* = .01); psychiatric-specific interventions including ECT (AOR = 21.10; *p* < .001) and mechanical restraints (AOR = 12.65; *p* = .004); and acute medical diagnoses (e.g., cardiac arrest, rhabdomyolysis, nephrolithiasis, and atrial fibrillation) (AOR = 8.56; *p* = .01) were associated with increased odds of VTE diagnosis (Table 3). Substance use was associated with decreased odds of VTE diagnosis (AOR = 0.14; *p* < .001).

**Table 3.** Factors independently associated with VTE, multivariable logistic regression.

	Adjusted OR (95% CI)	*p*-value
Demographics		
Age (years)	1.02 (0.98, 1.06)	0.44
Male	1.70 (0.35, 8.28)	0.51
Medical comorbidities		
Cancer	51.83 (3.66, 734.66)	0.004
Cardiovascular	7.83 (1.67, 36.69)	0.01
Insomnia	88.79 (3.48, 2265.47)	0.01
Substance use history	0.14 (0.03, 0.64)	<0.001
Psychiatric-specific interventions		
Electroconvulsive therapy	21.10 (3.90, 114.09)	<0.001
Mechanical restraints	12.65 (1.17, 136.59)	0.004
Acute physical health concerns		
Acute medical diagnosis	8.56 (1.95, 37.62)	0.01

OR: odds ratio; CI: confidence interval.

Wide confidence intervals reflect the limited sample size and should be interpreted cautiously.

## Discussion

This study is among the first conducted in Canada that investigated VTE occurrence and contributing factors in psychiatric inpatients. Overall, patients who experienced VTE tended to have more complex clinical profiles, including a greater burden of psychiatric and medical comorbidities, compared to those without VTE. However, these findings should be interpreted with caution due to the single-site design, relatively small sample size, and wide confidence intervals, which may limit the generalizability and precision of the results.

Factors for VTE at baseline were chronic comorbidities such as cardiovascular conditions and cancer. We did not identify biological sex as a factor related to VTE in this cohort of psychiatric inpatients which differs from the findings of a large population-based study which investigated the incidence of VTE among Australian hospitalized medical inpatients and found a higher rate of VTE in women.^15^ In our study, psychiatric interventions and acute medical diagnoses emerged as potential VTE-associated factors, which may indicate an exacerbating relationship between psychiatric and medical factors, irrespective of biological sex. Notably, more than two-thirds of VTE cases involved a PE, underscoring the potential severity of VTE among psychiatric inpatients.

Mechanical restraints were independently associated with VTE in our study which aligns with previous findings. The use of restraints has been associated with increased risk of DVT, longer hospital stays, more use of as needed medications, and the development of post-traumatic stress disorder.^16,17^ For psychiatric inpatients receiving treatment with mechanical restraints, a retrospective cohort study identified that restraints increased the risk of DVT by six-fold.^17^ Similarly, in a retrospective epidemiologic study of 39 psychiatric inpatients with diagnosed VTE events, those who restrained for over 24 hours had a 40.0% increased risk of VTE, compared to those not restrained.^2^ High prevalence of restraint-induced VTE suggest that the development of VTE is likely related to venous stasis and the implementation of periodic mobilization may help mitigate this phenomenon.^18^ Furthermore, the use of mechanical restraints should be limited to exceptional situations due to the associated risks and concerns about potential violations of human rights.^19^

ECT was another psychiatric-specific intervention independently associated with VTE in our study. In a case series of five psychiatric inpatients receiving ECT for catatonia, two patients (40.0%) were diagnosed with proximal DVTs, of which, one patient (20.0%) developed a PE following ECT.^6^ Similarly, a case report described a psychiatric inpatient undergoing ECT for major depressive disorder (MDD) who had an undiagnosed proximal DVT which embolized into a PE following four sessions of ECT.^20^ Although these patients had known VTE risk factors, particularly immobilization, these cases raise the possibility that use of VTE RAMs prior to ECT could help to identify high-risk patients who may benefit from thromboprophylaxis. It is important to note that ECT itself may not be the primary risk factor; rather, the underlying conditions for which ECT is administered such as catatonia, severe depression with psychomotor retardation, and/or dehydration due to poor oral intake, may independently elevate the risk of VTE. In the present study, catatonia was more commonly observed in patients with VTE compared to those without (15.6% vs 1.9%, respectively).

However, in our study, the association between catatonia and VTE was significant in the univariate analysis, but not in the multivariable logistic regression analysis. Catatonia may include abnormal posturing, muscular rigidity, and reduced motor activity which would increase VTE risk due to venous stasis.^3,21^ Current literature supports catatonia as a psychiatric-specific risk factor for developing VTE due to the lack of spontaneous movements, with one study reporting 61.1% of catatonic patients developed VTE.^2^

Known risk factors for VTE include acute medical complications and the findings of our study align with this well-established relationship. Acute medical complications are characterized by dysregulated immune networks that are associated with coagulation, including endothelial activation, hemostasis imbalance, production of neutrophil extracellular traps, and platelet activation.^22^ In our study, the number of diagnosed VTE cases tripled during the COVID-19 pandemic (4/year; 2019–2021) compared to pre-pandemic (1.16/year; 2007–2018) which suggests that COVID-19 may have induced a hypercoagulable state.^23^ Alternatively, the social distancing precautions during the COVID-19 pandemic may have decreased patient mobility on the inpatient unit, subsequently increasing VTE likelihood. Similarly, a systematic review and meta-analysis identified a pooled VTE incidence of 1.8% among hospitalized COVID-19 patients, which was 2-to-5 fold higher than the pre-pandemic population.^24^

Substance use, including opioids, benzodiazepines, cannabis, alcohol, and stimulants, appeared inversely associated with VTE (AOR = 0.14); however, this finding requires cautious interpretation due to potential confounding. This finding contrasts with existing literature, which has shown that opioid use disorder is associated with increased risk of VTE, particularly postoperatively.^25,26^ The discrepancy may be explained by several factors. Substance use was recorded as a single, broad variable, combining current use of stimulants and central nervous system (CNS) depressants, which may have masked substance-specific effects. Additionally, the high prevalence of substance among control patients could have influenced the association. It is also possible that patients with stimulant use disorder and/or experiencing withdrawal effects from CNS depressants exhibited increased motor activity, which may have reduced VTE risk. Furthermore, individuals presenting with substance use concerns may have had shorter hospitalizations.

To our knowledge, there is no standardized approach for VTE prophylaxis in psychiatry. The development of resources targeted at thromboprophylaxis for at risk patients, including staff education and RAMs, may help identify patients at risk for VTE and those who could benefit from anticoagulation.^12^ There has been some work published that add psychiatric-specific risk factors to pre-existing VTE RAMs. For instance, a multicenter cross-sectional study adapted and implemented a United Kingdom Department of Health VTE RAM into three inpatient psychiatric sites and identified that nearly one-third of patients were at increased risk of VTE.^27^ A research team in Japan developed a VTE RAM specifically for psychiatric admissions. This tool accounted for site-specific risk factors associated with VTE events, such as female sex, MDD, and catatonia. It demonstrated strong performance in identifying patients at risk of VTE, achieving an area under the curve of 0.816 (95% CI: 0.781–0.851).^11^ Ultimately, this research suggests that developing new screening tools can help identify at risk patients; however, a complete list of factors to include in these tools, and whether these factors differ between practice sites, is still unknown.

We recommend that all psychiatric inpatient settings implement a composite VTE prophylaxis RAM. This could involve using validated tools such as the Caprini or Padua Prediction RAMs to assess VTE risk.^28,29^ However, these tools do not account for psychiatric-specific VTE risk factors identified in this study, such as ECT and use of mechanical restraints. Therefore, these unique factors should be considered in conjunction with a validated RAM. The Caprini RAM assigns scores based on over 30 VTE risk factors, categorizing patients into risk levels with corresponding prophylaxis recommendations.^28^ For example, a 65-year-old female psychiatric inpatient with a body mass index greater than 25, on bed rest secondary to severe depression, receiving menopausal hormone therapy, and scheduled to receive ECT would likely be considered high risk. Based on the Caprini RAM and clinical judgment, this patient may benefit from thromboprophylaxis prior to ECT.

The limitations of this study are related to the retrospective single-site design. Data extraction was only as thorough as the information documented in the patient charts, resulting in some participants excluded due to incomplete data. Thirty-two patients diagnosed with acute VTE were identified, which is unclear if this is a true reflection of cases. Cases were identified between 2007 and 2021, while controls were selected based on being free of VTE at the end of the study period in 2021, rather than throughout the entire 2007 to 2021 period. This approach was taken to ensure that controls had not developed VTE by the end of follow-up, thereby reducing the risk of misclassification (i.e., inadvertently including future cases as controls). Controls were selected using a random sampling method from SCM. Notably, cases and controls were not matched on demographic or clinical variables such as age, sex, or medication use. Instead, potential confounding factors were accounted for thorough statistical adjustment in the analysis. The 15-year inclusion period is relatively long, during which treatment practices and healthcare settings may have changed. These temporal shifts were not accounted for and may have influenced control selection or affected the comparability of cases and controls over time, potentially introducing bias. Anticoagulants (e.g., warfarin and apixaban) may have been used for unrelated comorbidities, like atrial fibrillation, but may have also been treating asymptomatic VTE, potentially contributing to fewer VTE events. Additionally, the documentation of clinically relevant variables such as mobility level and compression stocking usage were inconsistent, making it difficult to analyze how these factors impacted the risk of VTE. This study has a potentially biased sample, as it was only conducted in a single hospital setting; thus, results may not be generalizable to other practice settings. The wide 95% CIs observed in the multivariable logistic regression analysis reflect the low number of events among cases, highlighting the need for cautious interpretation of the results. Furthermore, the limited sample size constrained the ability to conduct subgroup analyses, thereby hindering a more comprehensive understanding of the influence of multiple factors on individual patients.

## Conclusion

The primary aim of this study was to characterize the factors associated with VTE development in psychiatric inpatients. Further investigation is warranted with a larger sample size and multicenter study design with comparable care settings, as a clearer understanding of VTE risk factors and assessing the benefits and risks of thromboprophylaxis in psychiatric inpatients may contribute to the improvement of clinical outcomes. These findings underscore the need for tailored VTE prophylaxis strategies and RAMs adapted for all psychiatric inpatient settings including in Australasia.

## Data Availability

Deidentified data can be shared with others to support Open Science.*
